# AgriTrust—A Trust Management Approach for Smart Agriculture in Cloud-based Internet of Agriculture Things

**DOI:** 10.3390/s20216174

**Published:** 2020-10-29

**Authors:** Kamran Ahmad Awan, Ikram Ud Din, Ahmad Almogren, Hisham Almajed

**Affiliations:** 1 Department of Information Technology, The University of Haripur, Haripur 22620, Pakistan; kamranawan.2955@gmail.com (K.A.A.); ikramuddin205@yahoo.com (I.U.D.); 2 Department of Computer Science, College of Computer and Information Sciences, King Saud University, Riyadh 11633, Saudi Arabia; 438105079@student.ksu.edu.sa

**Keywords:** Internet of Agriculture Things, trust management, privacy, smart irrigation system, urbanization, digital innovation

## Abstract

Internet of Things (IoT) provides a diverse platform to automate things where smart agriculture is one of the most promising concepts in the field of Internet of Agriculture Things (IoAT). Due to the requirements of more processing power for computations and predictions, the concept of Cloud-based smart agriculture is proposed for autonomic systems. This is where digital innovation and technology helps to improve the quality of life in the area of urbanization expansion. For the integration of cloud in smart agriculture, the system is shown to have security and privacy challenges, and most significantly, the identification of malicious and compromised nodes along with a secure transmission of information between sensors, cloud, and base station (BS). The identification of malicious and compromised node among soil sensors communicating with the BS is a notable challenge in the BS to cloud communications. The trust management mechanism is proposed as one of the solutions providing a lightweight approach to identify these nodes. In this article, we have proposed a novel trust management mechanism to identify malicious and compromised nodes by utilizing trust parameters. The trust mechanism is an event-driven process that computes trust based on the pre-defined time interval and utilizes the previous trust degree to develop an absolute trust degree. The system also maintains the trust degree of a BS and cloud service providers using distinct approaches. We have also performed extensive simulations to evaluate the performance of the proposed mechanism against several potential attacks. In addition, this research helps to create friendlier environments and efficient agricultural productions for the migration of people to the cities.

## 1. Introduction

**Figure 1:**
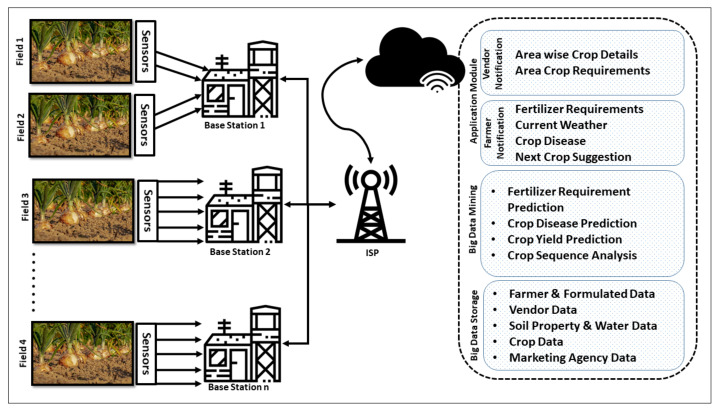
The architecture of cloud-based Internet of Agriculture Things (Adapted from [46]).

Internet Things (IoT) [1] provides a diverse opportunity to automate distinct domains, which include wireless sensor networks [2,3], home appliances [4,5], smart cities [6,7,8], healthcare [9,10,11,12,13], security and surveillance [14,15,16], energy consumption [17,18,19], agriculture automation [20], and many more [21,22,23,24,25]. The concept of a smart irrigation system [26] is proposed in the Cloud-based Internet of Agriculture Things (IoAT) [27] in which sensors gather the findings of soil and transmit it towards the base station (BS) to take the required actions [28]. The major applications of IoT in smart automated agriculture include agricultural monitoring and control [29], controlled environment agriculture [30], open-field agriculture [31], livestock applications [32], food supply chain tracking [33], IoT middleware and interoperability [34,35], multi-layer deployments [36], and commercial solutions [37]. This shows the important role of digital innovation and technology that help in delivering high quality services to improve the urban expansion and migration to cities. Our work helps to create friendlier environment and efficient agricultural productions that meet the demands of the migration of people to the cities. The architecture of cloud-based IoAT consists of agriculture fields equipped with sensors, BS, Internet, and cloud service providers. The sensors in agriculture can be of different types such as soil moisture [38], yield monitoring [39], water and environmental sensing [40], and soil micro-nutrient sensing among others [41,42,43]. These sensors can transmit the findings time-to-time towards the BS known as *in-field communications* performed by utilizing a WiFi Module (ESP8266) [44]. When the BS receives the findings, then it is independent to take few decisions, such as water supply, while other decisions can be taken with the help of cloud services, as shown in Figure 1. When the BS is required to transmit information towards the cloud for further computation and processing, then it utilizes the wireless connectivity provided by Internet Services Providers (ISP) [45] to transmit data. The services provided by clouds can be summarised as an application module that is further divided into farmer and vendor notifications, big data mining, and storage. The farmer notification includes the requirement of fertilizers, decisions required according to the weather, and crop diseases. The cloud service also informs the farmers about a suitable next crop agriculture. The vendor notification of the application module includes area wise crop details and requirements. The next service provided by clouds is big data mining in which it predicts the requirements of fertilizers, crop diseases, crop yields, and crop sequences. These predictions are performed by big data mining to adopt precautionary measures for better production. The most important service provided by the cloud is big data storage wherein farmers can store and formulate data, while the vendors along with marketing agencies can also save their data.

In IoAT, there are numerous security and privacy challenges that are required to be addressed in urban environment. Without a robust solution, it is not possible to take a complete advantage of the smart agriculture. The major security challenges of IoAT can be summarized as the identification of malicious and compromised nodes [47,48], authenticity [49], confidentiality [50,51], and privacy preservation of users [52]. In IoT, sensors are considered as edge nodes, which do not have enough capability to maintain resilience towards several potential attacks in distinct scenarios, such as smart homes [53], smart universities, and smart agriculture [54]. In all these environments, it is significant to keep resilience toward attacks generated by hackers with the help of malicious and compromised nodes. Besides, specifically in smart agriculture, if any of the sensors becomes malicious, then it affects the decision making that directly disturbs the production. Several existing approaches address the identification of malicious nodes by utilizing trust, however, the field of smart agriculture is totally neglected in those proposals. In this article, a privacy-aware trust management mechanism is proposed for smart agriculture, which provides security by identifying malicious and compromised nodes to maintain a secure environment. The proposed mechanism utilizes distinct trust parameters to evaluate the degree of trust between the sensors and BS, and then between the BS and the cloud. To improve the scalability and lighten the computational burden, AgriTrust is a time-driven approach that computes trust for a specific time and performs communications based on its computed trust degree. To improve the robustness and eliminate non-repudiation, the proposed mechanism uses the previous and current trust degrees to evaluate the aggregated trust while computing direct trust values. When the BS has no previous observation or previous trust, then it relies on indirect trust, which is computed based on recommendations. Each node has to maintain its minimum degree of trust that satisfies the threshold value to perform and transmit data.

The structure of the rest of the article is as follows: Section 2 elaborates on the existing work; Section 3 explains the proposed AgriTrust mechanism along with its working, trust composition, trust aggregation, and trust development; Section 4 presents the simulation results; and Section 5 concludes the paper.

## 2. Literature Review

The implementation of Cloud-based IoAT is not possible without addressing all the security challenges along with trust management, reliability, stack challenges, quality-of-service (QoS), and access control. Many current researches have pointed out numerous research gaps that are required to be addressed wherein trust management is one of those domains in which a significant amount of research is needed to provide adequate security. However, it has been neglected and no such mechanism is proposed to identify malicious nodes based on trust parameters. A considerable amount of research has been done in various fields of smart agriculture, which is elucidated in this section.

In [55], a study has been published that focuses on the digitization of smart cites and agriculture. The study stated that it is significant to use arrowhead technologies that increase the performance of a network and helps to address the challenges associated with transmission speed, latency, etc. With respect to smart agriculture, the study has integrated the arrowhead local cloud that consists of data connector components and data transformation engine connected with data storage and decision support units. The collected data is further used by data visualization component for aggregation and analytics. A smart irrigation system is proposed to efficiently utilize the energy resources [56]. The system designs a node having the capability of long-transmission and utilizes low energy by using SOC CC1310. Further, the proposed mechanism integrates 6LoWPAN along with fuzzy to predict the irrigation strategies.

In [57], another smart irrigation system is proposed that focuses on reducing the cost by using Message Queue Telemetry Transport protocol (MQTT). In the proposed mechanism, the system designs a simple water pumping mechanism controlled by sensors along with NodeMCU-12E and Esp8266. The transmission and receiving of sensor information is forwarded by utilizing MQTT protocol. The soil moisture sensor has to transmit the findings towards NodeMCU-12E, whereby it further takes the required actions. Another smart irrigation system is proposed to reduce the cost of irrigation by using low-cost moisture sensors along with XBEE communications [58]. The study stated that efficient water management plays a vital role and it should be the prime focus of the world to utilize it with precision to reduce water wastage. In the proposed mechanism, the soil moisture is gathered from the sensors where the XBEE communication is integrated in a centralized server that controls the water resources for supply when required.

In [59], a cryptographic algorithm is proposed to improve the security of IoT-based irrigation system. The study stated that it is important to provide security to those devices which have less resources where cryptography is one of the most prominent solutions to maintain the integrity and confidentiality among nodes. The approach utilizes Secure Hashing Algorithm (SHA-256), Rivest Cipher (RS4), and Elliptic-Curve Cryptography (ECC). In [60], a solar-based security system is proposed for the smart irrigation system in IoAT. The security is provided by using ARM LAC2148 with an integrated LASER and Solar panel along with the GSM module. The major contribution of the study is the use of ARM controller, which provides adequate accuracy with minimal error. Similarly, a secure watering mechanism was proposed for smart agriculture in [61], which uses fuzzy logic along with blockchain to provide security. The proposed approach uses an Android platform for the consumption of water in small or medium gardens. This approach uses sensors to capture data, which include moisture level of soil, air temperature, etc. The blockchain maintains the privacy and reliability where the fuzzy logic is used for decision marking.

Despite the fact that several existing approaches have been proposed for the smart irrigation system, a few of them are concerned about maintaining low-cost while others focus on the provision of energy-efficient systems. Moreover, the existing researches for security in IoAT require notable consideration to address several security challenges, such as data privacy, reliability, and integrity among others, which have been pointed out in [62,63,64,65,66]. Moreover, several trust management mechanisms are proposed in IoT showing to have a significant impact on the identification of malicious nodes [67,68]. While in IoAT, it is important to identify such nodes that can affect smart irrigation systems. In [69], the study discussed the adoption of IoT in smart agriculture and the role of trust to minimize the risk. The study elaborated the significance of trust and stated that trust is a key for the technology acceptance model, which can also play a vital role to build positive relations among nodes. However, trust management in IoAT is neglected over a decade and a robust mechanism is required for cloud-based IoAT to maintain a secure environment among sensors, BS, and cloud service providers.

## 3. Proposed AgriTrust Approach

A trust management mechanism is proposed as an alternative to traditional cryptography approaches that consume more energy where devices with less computational resources are unable to perform such computations that cause vulnerabilities. The smart agriculture in IoAT consists of several sensors placed in soil to collect and transmit information, while the BS and cloud perform several distinct computational operations to find the results and take the required actions. The identification of malicious and compromised sensors is a significant challenge because a malicious sensor can transmit wrong findings that affect the production of crops or can also execute potential attacks to reduce the performance of the BS. Moreover, the BS to cloud transmission faces the same challenges and it is important to maintain a secure environment, which is efficient to identify the malicious and compromised nodes while using less energy resources. To address these challenges, trust is the most prominent solution that has been neglected in IoAT till date. In this article, a trust management mechanism is proposed to identify malicious nodes and maintain a robust environment. The proposed approach includes three distinct trust management models, i.e., sensors to BS trust computation, cloud to BS trust computation, and BS to cloud computation, where all these computations use their own trust parameters to identify malicious and compromised nodes. The AgriTrust mechanism computes the trust based on direct computations. The direct trust evaluation utilizes the available observations related to pre-defined parameters and computes the trust. The computation of trust degree is performed by utilizing the statistical model, while the trust is computed by the centralized authorities, i.e., BS and cloud service providers. The trust computations performed by the authorities are time-driven, which means that when a degree of trust is computed, then the central authority has to compute the trust degree over a specific period of time.

**Figure 2:**
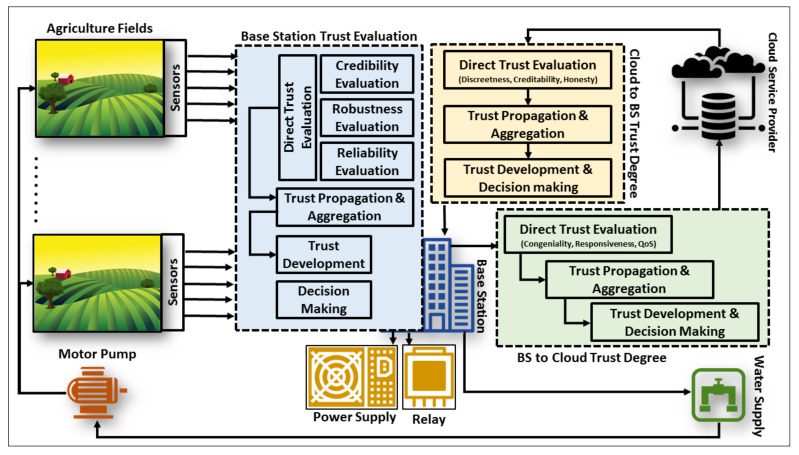
The AgriTrust architecture.

The proposed architecture of AgriTrust consists of agriculture fields equipped with sensors, dedicated BS, and cloud, as shown in Figure 2. The sensors collect data about the soil and transmit it to the BS. The communication between sensors and BS is equipped with WiFi module ESP8266 wireless connectivity. The sensors utilize in this model are soil moisture sensors that collect real-time data for water management. The yield monitoring sensor helps to identify spatial distribution of crop utilized for long term decision making. Other components are water, environmental, and soil micro-nutrients sensors. When a BS receives the data transmitted by the sensors, it first computes the trust value based on the pre-defined parameters and then compares it with the threshold value. If the trust degree meets the minimum requirement of trust, it accepts the value and exerts the required actions. On the other hand, if the requirements are not satisfied, then the BS neglects the value and starts monitoring that particular sensor for abnormal behaviors. The trust computation between the BS and cloud is also calculated based on the pre-defined parameters while utilizing the previous trust degree to evaluate the aggregated trust. The trust computations of agriculture sensors, BS, and cloud are elaborated in Section 3.1, Section 3.2, and Section 3.3, respectively. The relevant authority stores each computed observation in its particular category to fetch it for future computations. These observations are the previous computed trust degrees used by a node to formulate the aggregated trust value. The trust degree lies between 0.0 to 1.0, which means that 0.0 is the minimum trust and 1.0 is the maximum trust, whereas the default trust value for sensors is 0.5 and 0.6 for the BS and cloud, respectively. The trust computation in AgriTrust is time-driven and the default trust duration of superior trust per computation is 90 min, while it is 45 min in case of mid trust degree. When the BS computes the trust degree of a particular sensor or the cloud computes the trust degree of a BS, then they can communicate with each other for 90 min based on the same trust. On the contrary, if they want to exchange information after that specific time, they have to compute the trust degree again by using the same procedure.

### 3.1. Base Station to Sensor Trust Evaluation

The trust degree evaluation by the BS towards on-field sensors is initiated when they receive the transmitted data from the sensors. The sensors collect the findings and transmit data towards the BS. The BS evaluates the trust degree of that particular sensor first and then accepts the data. The trust evaluation of BS towards sensor is time-driven, which means that the BS evaluates the trust degree and accepts the data using the same trust for a specific period, as elaborated in Section 3. The trust evaluation process of sensors is illustrated in Algorithm 1 that is started by collecting the observation to identify whether it is possible to evaluate direct trust or should rely on the indirect/default trust degree. The trust parameters consist of credibility *(cr)*, robustness *(ro)*, and reliability *(re)*. Equation (1) illustrates the observation check of the BS-to-sensors trust evaluation where *p* represents the previous trust and *cr*, *ro*, *re* represent the trust parameters, while $ob_{1-n}^{cr}$, $ob_{1-n}^{ro}$, $ob_{1-n}^{re}$ show the number of available observations.

(1a) $$\begin{array}{c} P_{t}^{cr}=ob_{1}^{cr}+ob_{2}^{cr}+\ldots +ob_{n}^{cr} \end{array}$$

(1b) $$\begin{array}{c} P_{t}^{ro}=ob_{1}^{ro}+ob_{2}^{ro}+\ldots +ob_{n}^{ro} \end{array}$$

(1c) $$\begin{array}{c} P_{t}^{re}=ob_{1}^{re}+ob_{2}^{re}+\ldots +ob_{n}^{re} \end{array}$$

**Algorithm 1** Base Station to Sensor Direct Trust
1:**procedure** Observation Gathering($D_{ob}$)2:    $P_{t}^{cr}=ob_{1}^{cr}+ob_{2}^{cr}+\ldots +ob_{n}^{cr}$3:    $P_{t}^{ro}=ob_{1}^{ro}+ob_{2}^{ro}+\ldots +ob_{n}^{ro}$4:    $P_{t}^{re}=ob_{1}^{re}+ob_{2}^{re}+\ldots +ob_{n}^{re}$5:    **if** ($D_{ob}==Yes$) **then**6:        Compute Direct Trust;7:    **else**8:        Compute Indirect Trust;9:**procedure** Direct Trust Evaluation($D_{trust}^{n-id}$)10:    $c_{cr}^{trust}=\sum \left[ob_{1}^{cr}+ob_{2}^{cr}+\ldots +ob_{n}^{cr}\right]$11:    $c_{ro}^{trust}=\sum \left[ob_{1}^{ro}+ob_{2}^{ro}+\ldots +ob_{n}^{ro}\right]$12:    $c_{re}^{trust}=\sum \left[ob_{1}^{re}+ob_{2}^{re}+\ldots +ob_{n}^{re}\right]$13:**procedure** Trust Development($T_{abs}^{dev}$)14:    $T_{n-id}^{current}=\sum_{0.0}^{1.0}\left[c_{cr}^{trust}+c_{ro}^{trust}+c_{re}^{trust}\right]$15:    $T_{n-id}^{pre}=\sum \left[T_{n-id}^{pre_{1}}+T_{n-id}^{pre_{2}}+\ldots +T_{n-id}^{pre_{n}}\right]$16:    $abt_{n-id}^{agg}=T_{n-id}^{current}+T_{n-id}^{pre}$17:    $Agbt_{n-id}^{agg}=\phi \left[\sum_{0.0}^{1.0}abt_{agg}^{n-id}\right]$18:**procedure** Decision Making(ϕ(t))19:    $\phi \left(t\right)=\left\{\begin{array}{c} Sup-Trust\&\;\mathrm{if}\;t\geq 0.8\\ Mid-Trust\&\;\mathrm{if}\;t\geq 0.6\\ Def-Trust\&\;\mathrm{if}\;t\overset{\underset{\mathrm{def}}{}}{=}0.5\\ No-Trust\&\;\mathrm{if}\;t\leq 0.4 \end{array}\right.$20:    **if** (ϕ(t)≥0.6) **then**21:        DeclaredTrustworthy;22:    **else**23:        StartMonitoring;


If the observations are available about a particular sensor, then the AgriTrust starts evaluating the direct degree of trust by evaluating credibility, as represented in Equation (2). The credibility of a sensor shows the believability consist of subjective and objective components of trust. Equation (2) is evaluated by applying the summation function to all the available observations that give an output lies between the limit of the trust degree.

(2) $$c_{cr}^{trust}=\sum \left[ob_{1}^{cr}+ob_{2}^{cr}+\ldots +ob_{n}^{cr}\right]$$

In Equation (2), *c* represents current trust, *cr* is the credibility evaluation, and $ob_{1-n}^{cr}$ shows the number of available observations related to credibility. The next process is to evaluate the robustness of a particular sensor evaluated on the basis of quality of being rigorous against potential attacks. Equation (3) shows the evaluation of robustness by applying summation to the available observations, where *c* represents current trust, *ro* is the trust parameter of robustness, and $ob_{1-n}^{ro}$ shows the number of available observations at particular time (t).

(3) $$c_{ro}^{trust}=\sum \left[ob_{1}^{ro}+ob_{2}^{ro}+\ldots +ob_{n}^{ro}\right]$$

The last parameter evaluation is reliability, which represents the quality of being trustworthy and working consistency of a particular sensor. The trust evaluation of reliability is represented by Equation (4), where *re* represents the reliability parameter and $ob_{1-n}^{re}$ shows the observations related to reliability.

(4) $$c_{re}^{trust}=\sum \left[ob_{1}^{re}+ob_{2}^{re}+\ldots +ob_{n}^{re}\right]$$

After evaluating the trust parameters based on the available observations, the next process is to develop an absolute value of trust from all trust parameters evaluation. The process of trust development begins by applying summation to the evaluation of the current parameters, as represented in Equation (5). The equation also applies a limit to formulate the value lying in the trust threshold value to compare it during the decision making phase.

(5) $$T_{n-id}^{current}=\sum_{0.0}^{1.0}\left[c_{cr}^{trust}+c_{ro}^{trust}+c_{re}^{trust}\right]$$

In Equation (5), $T_{n-id}^{current}$ represents the current trust of a particular node, where *n-id* shows the unique identify of that node. The $c_{cr}^{trust}$, $c_{ro}^{trust}$ ,$c_{re}^{trust}$ represent the current trust evaluation of credibility, robustness, and reliability of trust parameters, respectively. After the development of trust parameter, the next process is to find the aggregate trust by utilizing the previous trust value that may provide an aggregated trust, as shown in Equation (6).

(6a) $$\begin{array}{c} T_{n-id}^{pre}=\sum \left[T_{n-id}^{pre_{1}}+T_{n-id}^{pre_{2}}+\ldots +T_{n-id}^{pre_{n}}\right] \end{array}$$

(6b) $$\begin{array}{c} Pabt_{n-id}^{agg}=T_{n-id}^{current}+T_{n-id}^{pre} \end{array}$$

After evaluating the aggregated trust, the BS compares it with the threshold value where t≥0.8 is the superior trust, t≥0.6 is the mid trust, while the default trust value is t≥0.5. If the trust degree of a sensor satisfies the threshold, then the BS accepts the findings of sensors and processes it for decision making. Otherwise, if the trust degree t≤0.4, then the BS neglects to accept the findings and starts monitoring sensors for any abnormal behavior. If the sensor trust degree is estimated as superior, then the BS will utilize and accept the findings by using the same trust degree for 90 min and 45 min if the trust value lies between 0.6–0.7.

### 3.2. Base Station to Cloud Trust Evaluation

In IoAT, the BS and cloud are independent to communicate and exchange information with each other at any time. Their communications required to be secure and it is significant to maintain the integrity of information exchange during communications to preserve the privacy. To address these challenges, it is significant to maintain the trustworthiness by evaluating the trust degree using trust parameters. The AgriTrust proposes a two-way mechanism to evaluate the trustworthiness. The BS trust evaluation towards the cloud is elaborated in this section, while the cloud service trust evaluation towards the BS is explained in Section 3.3. The trust evaluation flow process is illustrated in Figure 2, while the computation process is represented in Algorithm 2.
**Algorithm 2** Base Station to Cloud Trust Evaluation1:**procedure** Observation Gathering($D_{ob}$)2:    $P_{trust}^{cog}=ob_{1}^{cog}+ob_{2}^{cog}+\ldots +ob_{n}^{cog}$3:    $P_{trust}^{res}=ob_{1}^{res}+ob_{2}^{es}+\ldots +ob_{n}^{res}$4:    $P_{trust}^{qos}=ob_{1}^{qos}+ob_{2}^{qos}+\ldots +ob_{n}^{qos}$5:    **if** ($D_{ob}==Yes$) **then**6:        Compute Direct Trust;7:    **else**8:        Compute Indirect Trust;9:**procedure** Direct Trust Evaluation($D_{trust}^{c-id}$)10:    $c_{cog}^{trust}=\sum \left[ob_{1}^{cog}+ob_{2}^{cod}+\ldots +ob_{n}^{cog}\right]$11:    $c_{res}^{trust}=\sum \left[ob_{1}^{res}+ob_{2}^{res}+\ldots +ob_{n}^{res}\right]$12:    $c_{qos}^{trust}=\sum \left[ob_{1}^{qos}+ob_{2}^{qos}+\ldots +ob_{n}^{qos}\right]$13:**procedure** Trust Development($T_{abs}^{dev}$)14:    $T_{c-id}^{current}=\sum_{0.0}^{1.0}\left[c_{cog}^{trust}+c_{res}^{trust}+c_{qos}^{trust}\right]$15:    $T_{c-id}^{pre}=\sum \left[T_{c-id}^{pre_{1}}+T_{c-id}^{pre_{2}}+\ldots +T_{c-id}^{pre_{n}}\right]$16:    $abt_{c-id}^{agg}=T_{c-id}^{current}+T_{c-id}^{pre}$17:    $Agbt_{c-id}^{agg}=\phi \left[\sum_{0.0}^{1.0}abt_{agg}^{c-id}\right]$18:**procedure** Decision Making(ϕ(t))19:    $\phi \left(t\right)=\left\{\begin{array}{c} Sup-Trust\&\;\mathrm{if}\;t\geq 0.9\\ Mid-Trust\&\;\mathrm{if}\;t\geq 0.7\\ Def-Trust\&\;\mathrm{if}\;t\overset{\underset{\mathrm{def}}{}}{=}0.6\\ No-Trust\&\;\mathrm{if}\;t\leq 0.5 \end{array}\right.$20:    **if** (ϕ(t)≥0.5) **then**21:        DeclaredTrustworthy;22:    **else**23:        StartMonitoring;

The process of cloud trust evaluation started by collecting the observations of parameters, i.e., congeniality, responsiveness, and QoS. The congeniality represents the quality of congenial host and provides the services according to the requirements. The responsiveness parameter is evaluated based on the ability of the cloud to respond quickly and positively. The QoS is evaluated and graded based on transmit delay, overhead, and throughput. The evaluation of these parameters is illustrated in Equation (7). In Equation (7a), the evaluation of congenial trust is based on the available observation where *c* represents the current trust and $ob_{1-n}^{cog}$ shows the collection of available observations.

(7a) $$\begin{array}{c} c_{cog}^{trust}=\sum \left[ob_{1}^{cog}+ob_{2}^{cod}+\ldots +ob_{n}^{cog}\right] \end{array}$$

(7b) $$\begin{array}{c} c_{res}^{trust}=\sum \left[ob_{1}^{res}+ob_{2}^{res}+\ldots +ob_{n}^{res}\right] \end{array}$$

(7c) $$\begin{array}{c} c_{qos}^{trust}=\sum \left[ob_{1}^{qos}+ob_{2}^{qos}+\ldots +ob_{n}^{qos}\right] \end{array}$$

Equation (7b) shows the trust evaluation of responsiveness based on previous observations, while Equation (7c) represents the evaluation of QoS based on transmit delay, overhead, and throughput. In Equation (7b), *c, cog*, and *trust* show the congenial evaluation of current trust evaluated by applying the summation function on the current observations. After the evaluation of trust parameters, the BS will apply the summation to find the absolute value from the current values of trust, which can be computed using pre-defined parameters. The absolute trust development process is shown in Equation (8), where $T_{c-id}^{current}$ shows the development cloud trust, *c-id* is the unique identity of trust utilized by the BS to maintain and store the trust degree for future aggregation.

(8) $$T_{c-id}^{current}=\sum_{0.0}^{1.0}\left[c_{cog}^{trust}+c_{res}^{trust}+c_{qos}^{trust}\right]$$

The complete process of trust development leads the process to the next phase, which is used to compute the aggregated trust by applying summation to the previous trust value, as shows in Equation (9). Equation (9a) represents the summation of previous trust values where $T_{c-id}^{pre_{1-n}}$ shows the number of previous trust values that are available at specific time (t). Equation (9b) illustrates the computation of finding trust by adding the previous and current trust, while $T_{c-id}^{current}$ shows the current trust evaluation and $T_{c-id}^{pre}$ represents the previous trust estimation.

(9a) $$\begin{array}{c} T_{c-id}^{pre}=\sum \left[T_{c-id}^{pre_{1}}+T_{c-id}^{pre_{2}}+\ldots +T_{c-id}^{pre_{n}}\right] \end{array}$$

(9b) $$\begin{array}{c} abt_{c-id}^{agg}=T_{c-id}^{current}+T_{c-id}^{pre} \end{array}$$

After finding the aggregated trust, the BS compares it with the threshold value. If the absolute trust degree fulfils the requirement of the minimum trust value, then the BS will transmit and receive information for a specific period of time, as elaborated in Section 3.

### 3.3. Cloud to Base Station Trust Evaluation

The AgriTrust is a two way trust-evaluation mechanism in which the BS and cloud service providers can both maintain the trust between each other for secure communications. The transmission of data from the BS contains critical information and the cloud performs numerous operations on that data, as illustrated in Figure 1. After processing data, the cloud gets the results, which is then transmitted towards the BS for performing necessary operations. The cloud performs the trust evaluation of the BS to identify the trustworthiness for secure communications and integrity of data. The trust evaluation of cloud towards BS is a different process that begins by collecting the available observations about a particular BS. The process of trust degree computation is represented by Algorithm 3. The trust evaluation of the BS consists of discreetness, credibility, and honesty parameters.
**Algorithm 3** Cloud to Base Station Trust Evaluation1:**procedure** Observation Gathering($D_{ob}$)2:    $P_{trust}^{dis}=ob_{1}^{dis}+ob_{2}^{dis}+\ldots +ob_{n}^{dis}$3:    $P_{trust}^{cre}=ob_{1}^{cre}+ob_{2}^{cre}+\ldots +ob_{n}^{cre}$4:    $P_{trust}^{h}=ob_{1}^{h}+ob_{2}^{h}+\ldots +ob_{n}^{h}$5:    **if** ($D_{ob}==Yes$) **then**6:        Compute Direct Trust;7:    **else**8:        Compute Indirect Trust;9:**procedure** Direct Trust Evaluation($D_{trust}^{bs-id}$)10:    $c_{dis}^{trust}=\sum \left[ob_{1}^{dis}+ob_{2}^{dis}+\ldots +ob_{n}^{dis}\right]$11:    $c_{cre}^{trust}=\sum \left[ob_{1}^{cre}+ob_{2}^{cre}+\ldots +ob_{n}^{cre}\right]$12:    $c_{h}^{trust}=\sum \left[ob_{1}^{h}+ob_{2}^{h}+\ldots +ob_{n}^{h}\right]$13:**procedure** Trust Development($T_{abs}^{dev}$)14:    $T_{bs-id}^{current}=\sum_{0.0}^{1.0}\left[c_{dis}^{trust}+c_{cre}^{trust}+c_{h}^{trust}\right]$15:    $T_{bs-id}^{pre}=\sum \left[T_{bs-id}^{pre_{1}}+T_{bs-id}^{pre_{2}}+\ldots +T_{bs-id}^{pre_{n}}\right]$16:    $abt_{bs-id}^{agg}=T_{bs-id}^{current}+T_{bs-id}^{pre}$17:    $agbt_{bs-id}^{agg}=\phi \left[\sum_{0.0}^{1.0}abt_{agg}^{bs-id}\right]$18:**procedure** Decision Making(ϕ(t))19:    $\phi \left(t\right)=\left\{\begin{array}{c} Sup-Trust\&\;\mathrm{if}\;t\geq 0.9\\ Mid-Trust\&\;\mathrm{if}\;t\geq 0.7\\ Def-Trust\&\;\mathrm{if}\;t\overset{\underset{\mathrm{def}}{}}{=}0.6\\ No-Trust\&\;\mathrm{if}\;t\leq 0.5 \end{array}\right.$20:    **if** (ϕ(t)≥0.5) **then**21:        DeclaredTrustworthy;22:    **else**23:        StartMonitoring;

When the cloud receives data from the BS, it evaluates the trust and uses the same value for the pre-defined interval of time where the process of trust evaluation begins by evaluating the trust parameters of BS, which is illustrated by Equation (10). Equation (10a) shows the evaluation of BS discreetness where $T_{bs-id}^{current}$ represents the current trust evaluation of the BS with the utilization of unique identity represented by bs−id. After the evaluation of discreetness, the next process is to evaluate the credibility and honesty represented by Equations (10b,c).

(10a) $$\begin{array}{c} T_{bs-id}^{current}=\sum_{0.0}^{1.0}\left[c_{dis}^{trust}+c_{cre}^{trust}+c_{h}^{trust}\right] \end{array}$$

(10b) $$\begin{array}{c} c_{cre}^{trust}=\sum \left[ob_{1}^{cre}+ob_{2}^{cre}+\ldots +ob_{n}^{cre}\right] \end{array}$$

(10c) $$\begin{array}{c} c_{h}^{trust}=\sum \left[ob_{1}^{h}+ob_{2}^{h}+\ldots +ob_{n}^{h}\right] \end{array}$$

In Equation (10b), $c_{cre}^{trust}$ shows the evaluation of current trust degree of credibility by applying summation function to the available observations, where in Equation (10c), $c_{h}^{trust}$ shows the computation of BS honesty evaluation and $ob_{1-n}^{h}$ represents the number of available observations related to the honesty parameter. After the completion of trust parameter computation, the next step is to develop an absolute trust degree of the previous value and then aggregate these values with the current trust evaluation. In addition, it estimates the final trust degree of a particular BS to compare it with the threshold value to make the final decision. The computation of finding aggregated trust is represented by Equation (11).

(11a) $$\begin{array}{c} T_{bs-id}^{pre}=\sum \left[T_{bs-id}^{pre_{1}}+T_{bs-id}^{pre_{2}}+\ldots +T_{bs-id}^{pre_{n}}\right] \end{array}$$

(11b) $$\begin{array}{c} abt_{bs-id}^{agg}=T_{bs-id}^{current}+T_{bs-id}^{pre} \end{array}$$

(11c) $$\begin{array}{c} agbt_{bs-id}^{agg}=\phi \left[\sum_{0.0}^{1.0}abt_{agg}^{bs-id}\right]] \end{array}$$

In Equation (11a), $T_{bs-id}^{pre}$ represents the previous trust degrees of a particular node where bs−id shows the unique identity of a specific BS and $T_{c-id}^{pre_{1-n}}$ is the number of available trust degree. Equation (11b) demonstrates the evaluation of aggregated trust degree by adding the previous and current trust where $T_{bs-id}^{current}$ shows the current trust degree, while $T_{bs-id}^{pre}$ is the previous trust degree. The estimation of absolute trust degree by aggregating the previous and current trust leads the process to compare the final trust value with the threshold value. While the threshold value, in this case, is the same as elaborated during the BS-to-cloud trust degree evaluation in Section 3.2. If the trust degree satisfies the threshold value, then both will communicate. On the other hand, if the BS holds no trust, then the cloud will start monitoring for a pre-defined time. If the BS becomes trustworthy, then the cloud utilizes the same trust to communicate as trust computation, in this case, it is time-driven.

### 3.4. Indirect Trust Evaluation

The indirect trust is computed by gathering the recommendations from neighboring nodes. However, in the architecture of IoAT, the scenario is not the same. The architecture of IoAT includes three major components, i.e., sensors, BS, and cloud. As Sensor is only transmitting data to the BS and not communicating with anyone else, hence, gathering recommendations is not possible. In that situation when the BS cannot gather recommendations, it will assign the default degree of trust for communications. The situation of BS towards cloud is the same, however, the BS only transmits the data to a specific cloud and does not communicate with any other cloud or BS. Therefore, the recommendation gathering in this case is also not possible. Hence, the cloud assigns the default trust degree when it contains no previous observations. In the case of BS to cloud service provider, the BS can gather recommendations from the other BS as the cloud provides services to numerous BSs. In this situation, a particular BS generates a recommendation request for the neighboring connected BS and takes services from the same cloud. All BSs of the same cloud share recommendations based on their recommendations. When a BS receives these recommendations, then it applies the summation function to estimate the absolute trust degree, as shown in Equation (12).

(12) $$it_{c-id}^{bs-id}=\sum_{0.0}^{1.0}\left[rec_{1\to n_{th}}^{bs_{1}}+rec_{1\to n_{th}}^{bs_{2}}+rec_{1\to n_{th}}^{bs_{n}}\right]$$

After applying the summation function to the received recommendations, the BS will compare it with the predefined value and take the decision accordingly. However, there is no monitoring of cloud performed by the BS in case of low trust values.

## 4. Simulation and Results

This section discusses the simulation outcomes of the proposed AgriTrust mechanism under several scenarios. The performance of the BS and cloud service providers is also evaluated based on several QoS parameters. The simulations are performed in NS-3 simulator– an open-source discrete-event simulator. The number of nodes is varying under different situations where the trust degree lies between 0.0–1.0. The default trust degree of sensors is 0.5, while it is 0.6 in the case of cloud and BS. In the case of no trust, the normal monitoring time of sensors is 20 min, whereas if the BS contains no trust, then the monitoring time is 30 min. The trust computation in AgriTrut is an event-driven process and nodes use the same trust value for a specific interval of time without computing the trust whenever an event occurs. The nodes employ the computed trust value for a specific pre-defined period, i.e., 90 and 45 min in case of superior and mid-trust, respectively. The simulations are performed by varying the number of event-based trust computations and the results at 90 and 45 min are more reliable in comparison with other time-periods. The area minutes of one agriculture field is 245, 90, and 45 m^2^, and the number of nodes is varying from 50∼250 in a different scenario where the transmission rate is 8 megabits per second and the size of packets is varying from 10∼25 bytes.

### 4.1. Quality-of-Service Evaluation

**Figure 3:**
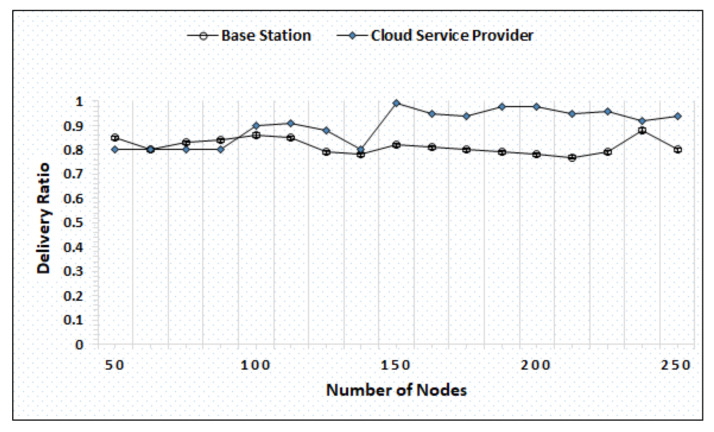
Delivery ratio evaluation by varying nodes.

The QoS evaluation is based on delivery ratio, latency, and overhead. The delivery ratio represents the number of packets successfully delivered from source to destination. The latency in the cloud-based IoAT causes the burden of computations on the BS or Cloud, while the overhead ratio represents the combination of indirect computational time. The QoS evaluation is performed under 4 distinct scenarios by varying the number of nodes from a minimum of 50 to a maximum of 250. Figure 3 represents the evaluation outcomes of the BS and cloud delivery ratios, which reveal that both maintain higher throughput. The BS maintains the average delivery ratio of 0.8, while the cloud sustains with 0.9. The cloud reaches higher delivery ratio, i.e., 0.99, when the number of nodes becomes 150. Whereas, the BS reaches the maximum delivery ratio when the number of nodes is near to 250.

**Figure 4:**
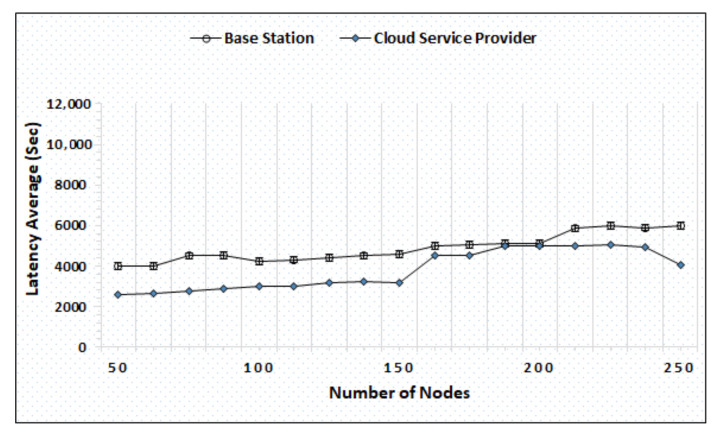
Latency average evaluation by varying nodes.

**Figure 5:**
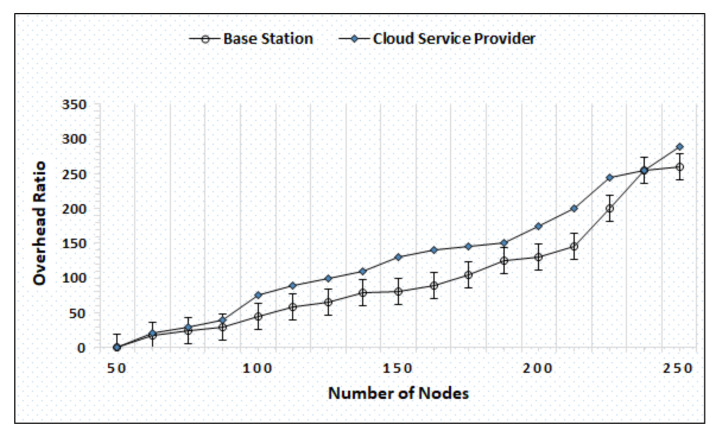
Overhead ratio evaluation by varying nodes.

Figure 4 represents the evaluation of latency average measured in time (seconds) by varying the number of nodes between 50–250, and the results show that the BS and cloud both successfully maintain the computational burden and compute the trust timely and respond back quickly. The minimum latency of the BS and cloud is 4000 and 2100, while the maximum latency is 5700 and 4260, respectively. Figure 5 shows the overhead ratio of the cloud and BS by increasing the number of nodes after a specific time. The simulation outcome shows the increase in the overhead ratios as the number of nodes increases with time.

### 4.2. Honest and Dishonest Precision Evaluation

**Figure 6:**
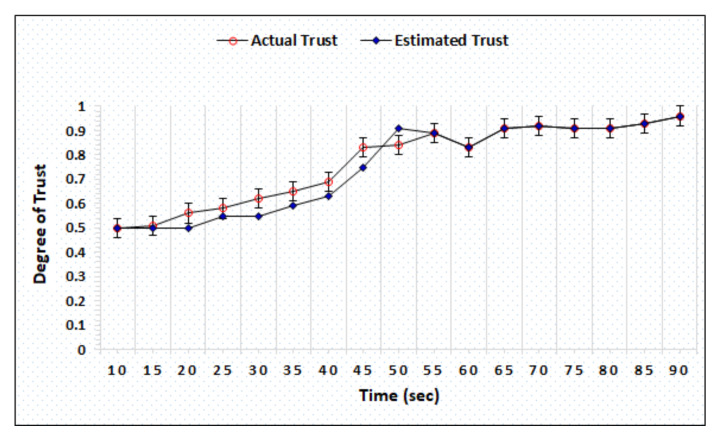
Trust degree of honest sensor.

**Figure 7:**
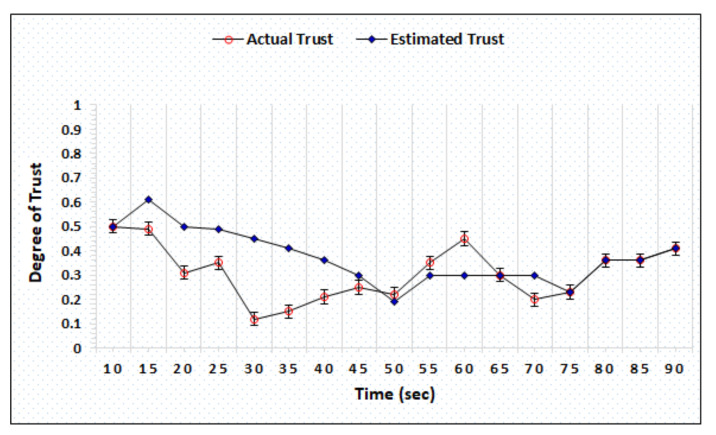
Trust degree of dishonest sensor.

The honest and dishonest trust precision represent the difference in the actual and estimated trust computed by AgriTrust. In the trust precision evaluation, the trust degrees of honest cloud, sensor, and BS have been simulated. Figure 6 represents the simulation outcomes of the trust degree of honest server, which shows that the proposed mechanism is able to estimate the actual trust within 55 s. Figure 7 shows the trust computation outcome of a dishonest sensor in which the AgriTrust is able to estimate the actual trust of a particular sensor within 75 s. The significant aspect of trust estimation is that the proposed approach assigns the low degree of trust in comparison to the actual trust of a node.

**Figure 8:**
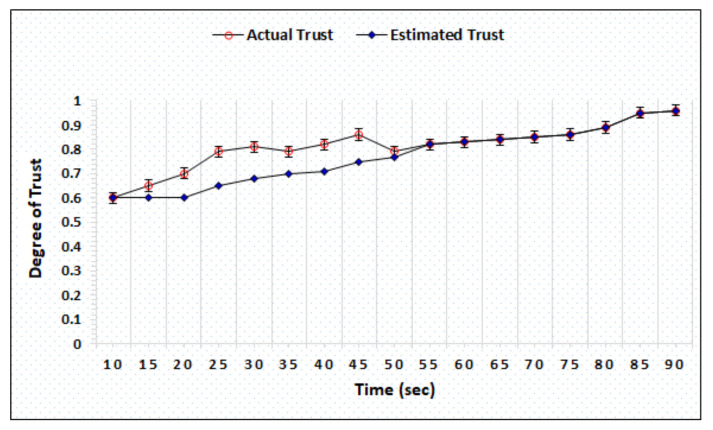
Trust degree of honest base station.

**Figure 9:**
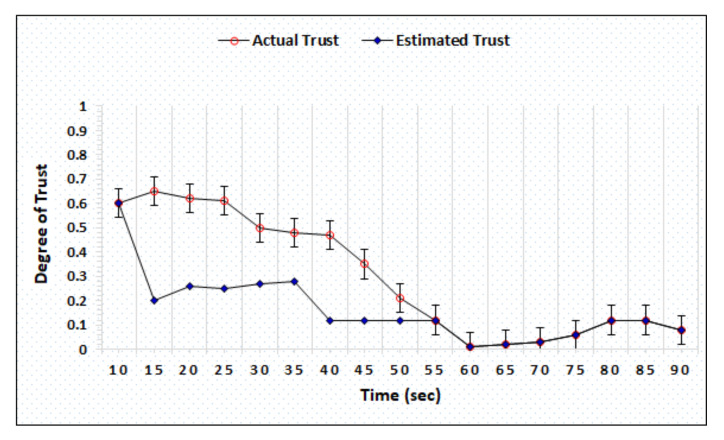
Trust degree of dishonest base station.

**Figure 10:**
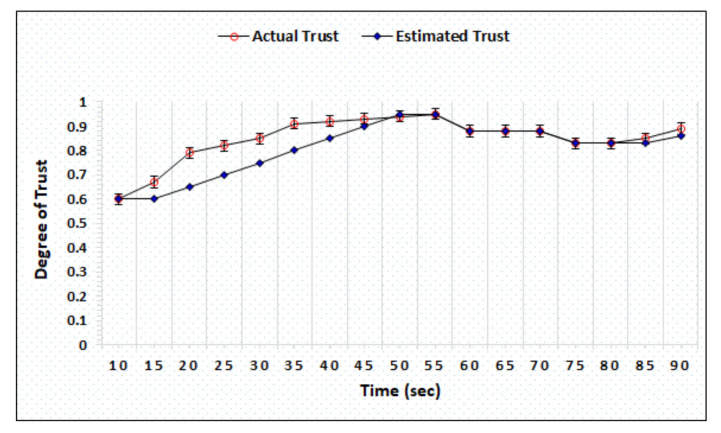
Trust degree of honest cloud.

**Figure 11:**
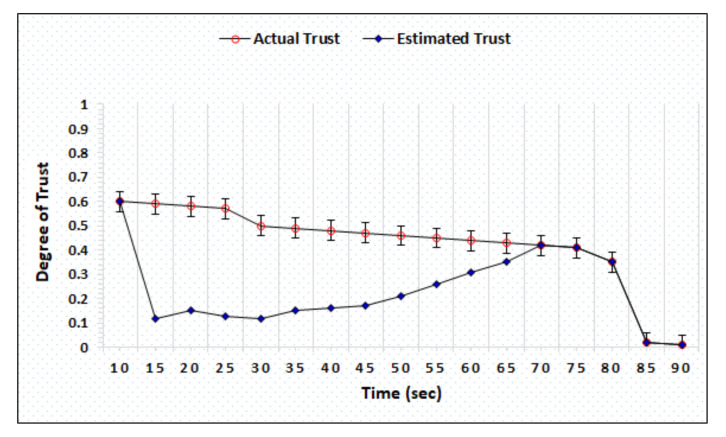
Trust degree of dishonest cloud.

Figure 8 represents the trust precision of honest BS evaluated by AgriTrust and the simulation outcome shows that the actual and estimated trust become similar after 55 s. In comparison to the trust degree of an honest BS, the AgriTrust takes 5 more seconds to estimate the actual trust of dishonest BS (see Figure 9). Further, we have also evaluated the trust precision of honest and dishonest cloud service providers to validate the performance of the proposed approach and the result shows that AgriTrust can estimate the actual trust degree of an honest node after 55 s. of separate computations over distinct time, as presented in Figure 10. Figure 11 shows the trust precision of dishonest cloud where the proposed approach can identify the actual trust degree after 70 s. The significant aspect of trust degree precision evaluation is that AgriTrust estimates and assigns the lower degree of trust in comparison to the actual trust.

### 4.3. Whitewashing Attack

**Figure 12:**
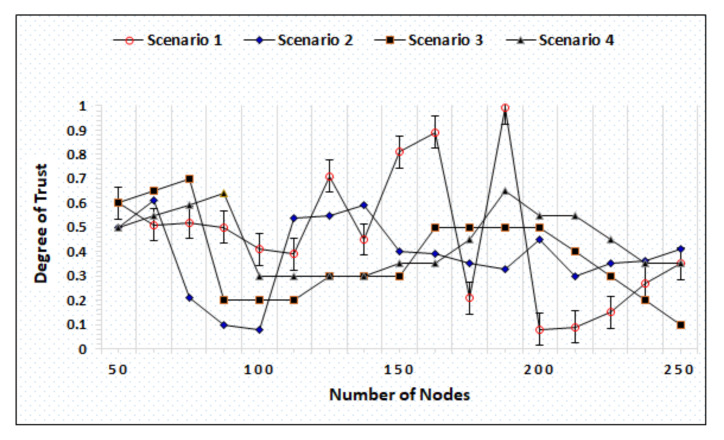
White washing attack with distinct scenario.

Whitewashing is a kind of attack whereby a node leaves the network with a low reputation and rejoins to gain the default reputation for communications. In the IoAT architecture, the most chances of executing a whitewashing attack are performed by the BS because sensors do not have the ability to leave the network. Similarly, it is also difficult for the cloud to leave the network as it is connected with numerous BSs. Figure 12 represents the simulation outcomes of the whitewashing attack under different scenarios created by increasing the number of nodes and the percentage ratios of malicious nodes. In scenario-1, the number of nodes variation made is 50∼250 with 20% malicious nodes where Figure 12 shows that the proposed mechanism assigns the lowest degree of trust after identifying malicious nodes and assigns higher/superior trust degree when it recognizes good/well-reputed nodes. In scenario-2, the total number of nodes is 50∼250 where 40% of the nodes are malicious and compromised. The simulation results show the effective performance of AgriTrust, which successfully identifies the nodes that try to execute the attack. In scenario-3, the variation of nodes is the same while the total percentage of malicious and compromised nodes increases as 60%. The simulation outcomes represent the efficient performance of the proposed methodology even with higher number of malicious nodes. In the last scenario, the number of node variations is also the same, but the number of malicious nodes becomes 80%. When the number of malicious nodes increases, it becomes difficult to maintain a secure and robust environment. In Figure 12, the result represents that AgriTrust successfully identifies the attacking nodes and maintains non-repudiation.

### 4.4. On-off Attack

**Figure 13:**
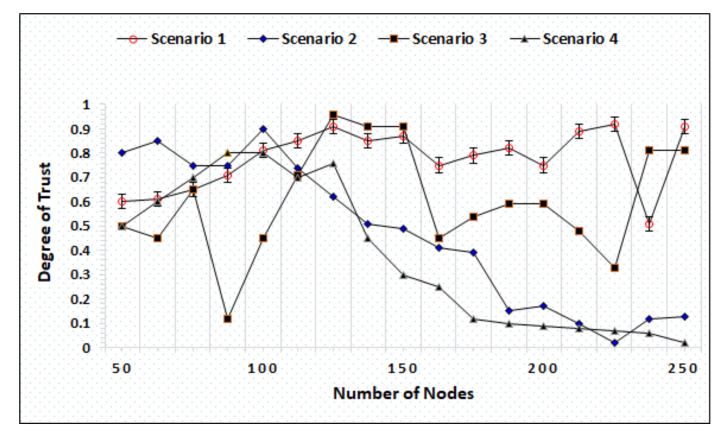
Comparison of AgriTrust against on-off attacks.

It is a type of attack in which nodes start behaving maliciously after receiving a low trust degree that affects the performance of the whole network. The successful execution of an on-off attack may compromise the network that can raise the integrity and privacy challenges in addition to affect the decision making at the cloud side, which directly affects the agriculture fields. Figure 13 represents the simulation results with four different scenarios where the BS becomes malicious and tries to execute an on-off attack. Whereas the cloud has the responsibility to identify and stop them by adding into the monitoring list. In four different scenarios, the number of node variations is from 50∼250 where the percentage of malicious nodes are 15%, 25%, 35%, and 45%, respectively. As illustrated in Figure 13, in scenarios 2 and 4, the maximum number of malicious nodes execute the attacks successfully identified by AgriTrust while showing a significant downfall in the trust degree when the number of nodes reaches 100. As mentioned earlier, the number of malicious nodes in scenario 1 is more limited and no node is able to execute the attack because the trust degree graph shows the highest trust degree assigned to the nodes. Scenario 3 represents notable fluctuations after each time interval showing the identification and assigning low trust degree every time when malicious nodes try to execute an attack.

### 4.5. Energy Consumption

**Figure 14:**
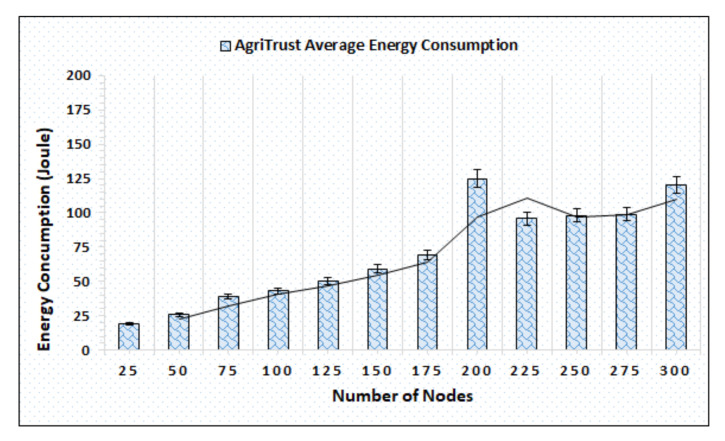
Energy consumption of AgriTrust w.r.t. nodes.

The energy consumption is significant for the successful implementation of Green IoAT. Also, there are numerous remote areas where a continuous supply of energy resources is not possible and they have to utilize the resources effectively and efficiently. Figure 14 illustrates the simulation results of the proposed mechanism, which shows the effective utilization of AgriTrust while performing trust computations. During simulations, the consumption of energy resources is represented by Joule whereby the simulation outcomes show that the computations performed by AgriTrust require fewer energy resources, which make it efficient for such remote areas where a continuous supply of energy is not possible. The effective utilization of energy resources makes AgriTrust a suitable approach for Green IoAT.

## 5. Conclusion

Smart automated agriculture is a significant concept presented in the domain of Internet of Agriculture Things (IoAT) wherein several sensors are placed in soil to monitor it and transmit the findings towards the BS and it further takes help from the cloud to make effective decisions to take perfect actions. However, security in the major components of smart agriculture requires a lightweight mechanism that can identify malicious and compromised nodes to maintain a secure environment. Moreover, one of the significant concepts of trust management is proposed by researchers that can play a notable role to maintain an adequate secure environment in IOAT, but it is neglected in the field of smart agriculture. In this article, a time driven-based trust management mechanism is proposed to identify those nodes which can affect secure environments of IoAT by any means. The proposed AgriTrust approach consists of three different trust management mechanisms, i.e., sensors to BS, cloud to BS, and BS to cloud trust evaluations. Each trust evaluation uses distinct trust parameters to identify malicious and compromised nodes. The simulation results illustrate the effective performance of these parameters in identifying malicious nodes where AgriTrust estimates the actual trust degree of nodes in a minimal time. The proposed work can be extended by employing artificial neural network to make the system intelligent having the capability to predict the malicious behavior of nodes.
